# *rad21* Is Involved in Corneal Stroma Development by Regulating Neural Crest Migration

**DOI:** 10.3390/ijms21207807

**Published:** 2020-10-21

**Authors:** Bi Ning Zhang, Yu Liu, Qichen Yang, Pui Ying Leung, Chengdong Wang, Thomas Chi Bun Wong, Clement C. Tham, Sun On Chan, Chi Pui Pang, Li Jia Chen, Job Dekker, Hui Zhao, Wai Kit Chu

**Affiliations:** 1 Department of Ophthalmology and Visual Sciences, The Chinese University of Hong Kong, Hong Kong, China; zbnxtt@gmail.com (B.N.Z.); yangqichen@link.cuhk.edu.hk (Q.Y.); clemtham@cuhk.edu.hk (C.C.T.); cppang@cuhk.edu.hk (C.P.P.); lijia_chen@cuhk.edu.hk (L.J.C.); 2 State Key Laboratory Cultivation Base, Shandong Provincial Key Laboratory of Ophthalmology, Shandong Eye Institute, Shandong First Medical University & Shandong Academy of Medical Sciences, Qingdao 266071, China; 3 Program in Systems Biology, Department of Biochemistry and Molecular Pharmacology, University of Massachusetts Medical School, 368 Plantation Street, Worcester, MA 01605, USA; YuSunny.Liu@umassmed.edu (Y.L.); job.dekker@umassmed.edu (J.D.); 4 School of Biomedical Sciences, Faculty of Medicine, the Chinese University of Hong Kong, Hong Kong, China; peggyleung@e.cuhk.edu.hk (P.Y.L.); wangcdg@hotmail.com (C.W.); wong2035@cuhk.edu.hk (T.C.B.W.); sunonchan@cuhk.edu.hk (S.O.C.); 5 Howard Hughes Medical Institute, 4000 Jones Bridge Road, Chevy Chase, MD 20815-6789, USA; 6 Key Laboratory for Regenerative Medicine, Ministry of Education, School of Biomedical Sciences, Faculty of Medicine, the Chinese University of Hong Kong, Hong Kong, China; 7 Kunming Institute of Zoology Chinese Academy of Sciences-the Chinese University of Hong Kong Joint Laboratory of Bioresources and Molecular Research of Common Diseases, Kunming 650223, China; 8 Joint Shantou International Eye Center, Shantou University and the Chinese University of Hong Kong, Shantou 515041, China

**Keywords:** *rad21*, neural crest migration, *Xenopus laevis*, corneal stroma

## Abstract

Previously, we identified RAD21^R450C^ from a peripheral sclerocornea pedigree. Injection of this *rad21* variant mRNA into *Xenopus laevis* embryos disrupted the organization of corneal stroma fibrils. To understand the mechanisms of RAD21-mediated corneal stroma defects, gene expression and chromosome conformation analysis were performed using cells from family members affected by peripheral sclerocornea. Both gene expression and chromosome conformation of cell adhesion genes were affected in cells carrying the heterozygous *rad21* variant. Since cell migration is essential in early embryonic development and sclerocornea is a congenital disease, we studied neural crest migration during cornea development in *X. laevis* embryos. In *X. laevis* embryos injected with *rad21* mutant mRNA, neural crest migration was disrupted, and the number of neural crest-derived periocular mesenchymes decreased significantly in the corneal stroma region. Our data indicate that the RAD21^R450C^ variant contributes to peripheral sclerocornea by modifying chromosome conformation and gene expression, therefore disturbing neural crest cell migration, which suggests RAD21 plays a key role in corneal stroma development.

## 1. Introduction

Peripheral sclerocornea is a rare congenital disorder characterized by opacification of the cornea rim. Previously, we identified a nonsynonymous RAD21^R450C^ point mutation in a peripheral sclerocornea pedigree spanning three generations [1]. Patients in this pedigree showed peripheral corneal opacity and significant decline in central corneal thickness, suggesting abnormalities in corneal stroma [1]. The injection of the corresponding mutant *rad21* mRNA into *Xenopus laevis* embryos disrupted the organization of corneal stroma fibrils [2], a phenotype that has been reported in sclerocornea patients [3]. While the uniformly lamellar layers of stromal fibrils contribute to corneal transparency, disorganization of these fibrils will cause corneal opacity [4]. We also found that the apex cornea was significantly thinner in family members affected by peripheral sclerocornea compared to unaffected family members [5]. Interestingly, a recent genome-wide association study (GWAS) reported that single-nucleotide polymorphisms (SNPs) in *WNT7B* are associated with central corneal thickness (CCT) in South Indian pedigrees [6]. In this study, the top *WNT7B* SNP s9330813 was reported to be in strong equilibrium with rs9723267, an SNP that was reported to disrupt RAD21 and CTCF binding sites [6]. This study suggested that there might be a link regulating RAD21-related transcription activity and CCT [6].

The lamellar layers of vertebrate corneal stroma are derived from neural crest cells (NCCs) [7,8]. During vertebrate embryogenesis, at the end of gastrulation, a specific cell population called neural crest is induced at the border of the neural plate and the non-neural ectoderm [9]. Soon after specification, NCCs undergo epithelial–mesenchymal transition (EMT) and become mobile [10]. The NCCs delaminate from the neural plate border and migrate to their appropriate location for subsequent differentiation. Among them, some mesenchymal cells migrate into the space between the lens vesicle and the surface ectoderm and finally differentiate into stromal keratocytes [11]. These keratocytes synthesize and secrete stromal collagens and proteoglycans to form corneal stroma extracellular matrix [12].

Besides mediating sister chromatid cohesion [13] and DNA damage repair [14,15,16], cohesin has been proposed to extrude DNA loops to establish chromosome structures like CTCF–CTCF loops and topologically associating domains (TADs) [17,18]. Somatic mutations in the cohesin complex have been identified in many diseases including cancer. For example, mutations in STAG2, a component of the cohesin complex, have been reported to be frequently associated with bladder cancers and leukemia [19,20]. Moreover, a recent study found that RAD21 mutations in children could lead to congenital cohesinopathy [21]. To explore whether RAD21^R450C^ could regulate chromosome organization and control the expression of genes that are important at the early stage of corneal development, RNA-Seq and Hi-C analyses were performed with human-derived lymphoblastoid cell lines (LCLs) obtained from our recently identified peripheral sclerocornea pedigree [2]. Hi-C is a method to study the three-dimensional architecture of whole genomes by coupling proximity-based DNA ligation with massively parallel DNA sequencing [22]. Since these sclerocornea pedigree members still possess functional vision, only peripheral blood rather than cornea tissues was collected for this study. As in Hi-C analysis, TADs have been reported to be highly conserved in various tissues and species [23,24], making the Hi-C results usefulto extend the knowledge we gained from LCLs to cornea. Our results show that the migration pathway of neural crest cells is disturbed by the RAD21^R450C^ mutation.

## 2. Results

### 2.1. Expression of Cell Adhesion Pathway Genes Is Altered in LCLs Isolated from Family Members Affected by Peripheral Sclerocornea 

To identify molecular signatures that are associated with peripheral sclerocornea, we performed RNA-Seq analysis on four LCLs derived from family members of the same generation of the peripheral sclerocornea pedigree, including two LCLs isolated from peripheral sclerocornea-affected family members (II-2 and II-3) and two LCLs from unaffected family members (II-4 and II-5). To validate the RNA sequencing results, the expression of top 5 up- and top 4 down-regulated genes were verified in the four LCLs selected for RNA-Seq and an additional LCL from the affected family member III-5. qPCR confirmed the increased expression of *SEPT10*, *PCDHGC3*, *RPS18*, *CCZ1*, *SLFN12L*, and *RPS3A* and the decreased expression of *GTSF1*, *IL32*, *ARHGAP44*, and *GOLGA8B* in affected members compared to unaffected family members (Supplementary Figure S1).

**Figure 1:**
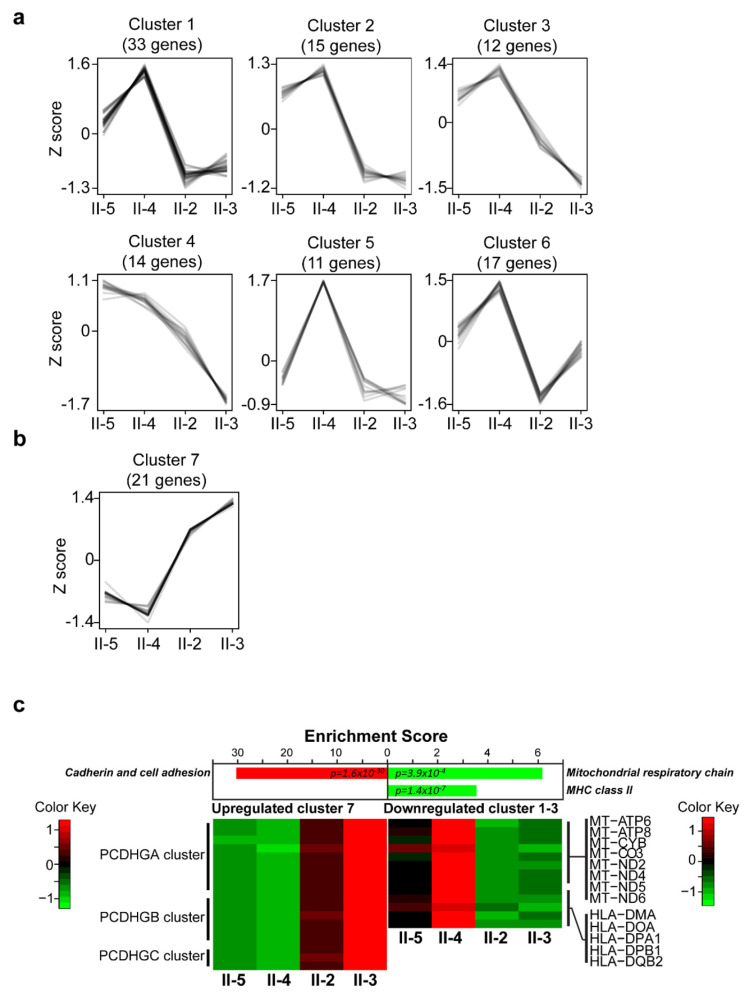
Clusters of expression profiles of differentially expressed genes (DEGs) and expression levels. (**a**) Six different expression clusters were obtained from downregulated genes. (**b**) One expression cluster was identified from the upregulated genes. (**c**) Expression level changes were detected in three functional groups in affected lymphoblastoid cell lines (LCLs). The expression levels of three *PCDHG* gene clusters (*PCDHGA*, *PCDHGB*, and *PCDHGC*) were upregulated in LCLs from affected members. Eight mitochondria-related genes and five MHC class II-related genes showed reduced expression levels in affected-members LCLs. The functional enrichment scores were obtained by using DAVID bioinformatics. Three significant functional groups with false discovery rate (FDR) < 0.05 were obtained, and the expression levels of genes within these groups are shown.

RNA-Seq analysis identified 362 coding genes that were differentially expressed (DEG) (fold changes less than 0.6 or greater than 1.66) in the LCLs isolated from affected compared to those from unaffected family members (Supplementary Table S1). These DEGs were expressed in at least one LCL [Reads Per Kilobase Million (RPKM) > 0.9], and their expression profiles displayed high variance when comparing LCLs isolated from affected members to those from unaffected members. Therefore, we clustered these DEGs based on their expression profiles across the four LCLs (Figure 1a,b). The downregulated genes in the LCLs derived from the affected family members were grouped into six clusters (Figure 1a and Supplementary Table S2), whereas the upregulated genes formed one cluster (Figure 1b and Supplementary Table S2). Downregulated gene cluster 1, 2, and 3 were pooled together as the downregulated group for further functional analysis, since they showed clear differential expression patterns in unaffected (II-4 and II-5) and affected groups (II-2 and II-3) (Figure 1a). Functional enrichment analyses were performed on both downregulated and upregulated groups. The upregulated genes showed function enrichments in cadherin and cell adhesion, indicating changes of cellular processes such as cell migration and cell contacts in LCLs from the affected member (Figure 1c). The downregulated genes appeared significantly associated with mitochondrial respiratory chain and MHC class II molecules (Figure 1c). Since MHC class II molecules are specific to immune cells and all LCLs were derived from B cells [25], we inferred that the affected LCLs presented altered mitochondria activity and cell surface molecules. In summary, our transcriptome analysis showed that the expression levels of cell adhesion genes were upregulated in LCLs from affected members.

### 2.2. The Genes Involved in Chromosome Organization of Cell Adhesion Pathways Are Altered in LCLs Derived from Family Members Affected by Peripheral Sclerocornea

RAD21 is a key component of the cohesin complex that plays important roles in both sister chromatin cohesion and chromosome organization [17,18,26,27]. We therefore examined whether cells expressing RAD21^R450C^ displayed altered chromosome organization and, if so, how this related to gene expression changes in the affected LCLs described above. We performed Hi-C analysis on the four LCLs, which we also used for RNA-Seq analysis. From our Hi-C results, we did not observe major changes in loops, TADs, and compartments (Supplementary Figure S2), indicating RAD21^R450C^ did not cause significant changes in global chromosome organizations in affected-members LCLs. Since R450C is a heterozygous variant in the affected LCLs [5], wild-type RAD21 may compensate the effects of RAD21^R450C^. To gain a more detailed view of the chromosomal spatial structural change, we thus measured the cumulative local chromosome contacts across each locus as insulation scores (IS) [28] and then calculated the insulation score changes (ISC) reflecting local chromosome organization changes [29]. We compared IS in the LCLs from unaffected and affected members to identify loci that displayed local chromosome structural changes induced by RAD21^R450C^. The comparison revealed that 7207 of 135,147 20 kb chromosome bins (5.3%) showed significant ISC between the affected and unaffected LCLs (bins with ISC beyond 95% confidence interval, 20 kb bins with ISC less than −0.15 or greater than 0.15), suggesting local chromosomal contact changes within these areas (Supplementary Table S3). Disruption of RAD21 or RAD21 loading/unloading causes compartment changes [26]. We thus obtained Eigenvector value (E1) as reflecting compartment signals and calculated E1 value changes (E1Cs) of each 200 kb bin across the genome. Among 15,490 of 200 kb bins, we found that 13,874 E1Cs and 502 E1Cs were significant when comparing the affected and the unaffected LCLs (E1C less than −0.172 or greater than 0.211, and beyond 95% confidence interval). We overlapped 13,874 E1C bins with 7207 significant ISC bins to identify 13,874 E1C bins containing 2049 significant ISC bins, while 154 significant ISC bins were located in 502 significant E1C bins, revealing significant enrichment of ISCs in these 502 E1Cs (Supplementary Table S4, Fisher Exact Test, *p* < 0.001). We then examined DEGs within the significant E1C bins and found no significant association of DEGs with significant E1Cs (Fisher Exact Test, *p* = 0.0676). Our results showed a strong association of significant ISC with significant compartment changes, indicating that RAD21^R450C^ causes both local insulation and compartment changes.

**Figure 2:**
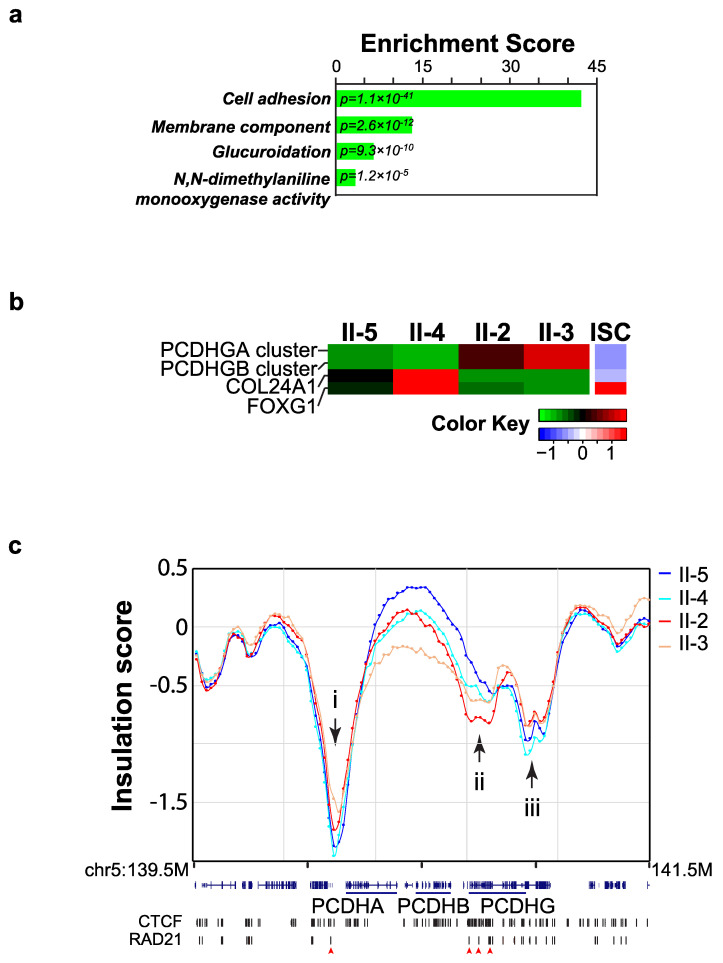
Local chromosome organization changes were detected in LCLs. (**a**) The coding genes overlapping with insulation score change (ISC) bins were chosen for functional enrichment analysis. Significant functional groups (FDR < 0.05) identified were cell adhesion, membrane components, glucuronidation, and *N*,*N*-dimethylaniline monooxygenase activity. (**b**) Local chromosome structural changes and gene expression changes in LCLs. Four differentially expressed genes/gene clusters showed changes in isolation scores, indicating local chromosome structural alterations may lead to expression changes in LCLs. (**c**) RAD21^R450C^ caused insulation score changes in *PCDH* gene clusters. Insulation score profile across the *PCDH* gene clusters. Both of these loci showed CTCF and RAD21 binding signals (indicated by red arrowheads) in GM12878 cells.

We then overlapped the significant 7207 ISC bins with coding gene loci and identified 1587 genes residing in 3943 ISC bins (54.7% of these significant ISC bins) (Supplementary Table S5). We performed a functional analysis of these 1587 ISC genes and found that the genes with decreased IS were associated with important functions, including cell adhesion, membrane structure, glucuronidation, and *N*,*N*-dimethylaniline monooxygenase activity (Figure 2a). Among these ISC genes, four genes or gene clusters, including two groups of protocadherin gamma (*PCDHG*) cluster subtype genes, a collagen-encoding gene (*COL24A1*), and a forkhead box transcription factor (*FOXG1*), were also DEGs (Figure 2b). This result indicated that local chromosome organization changes are correlated with altered expression levels of these DEGs. To illustrate their local chromosome structural changes, we generated IS profiles of the *PCDHG* gene clusters and integrated them with CTCF and RAD21 ChIPseq data from GM12878 cells, a cell line that was generated using the same protocol as that utilized our LCLs (Figure 2c) [30]. The *PCDHG* subclusters were located within two TADs, and the TAD boundaries are indicated by black arrows (i, ii, and iii, Figure 2c). CTCF and RAD21 ChIP signals were enriched within these three boundaries (Figure 2c, red arrowheads), consistent with previous reports that CTCF and RAD21 are enriched within TAD boundaries [31]. Though not very strong, there were insulation changes at the TAD boundaries between LCLs from the affected and the unaffected family members. In both affected LCLs (II-2 and II-3), the insulation of the first and third boundaries decreased (Figure 2c), whereas the insulation of the second boundary increased (Figure 2c, red and yellow dash lines). It has been reported that insulation changes within TAD boundaries can alter gene expression [32]. Therefore, insulation changes of these three TAD boundaries in affected-members LCLs could contribute to the expression alterations of the *PCDH* clusters. Similar changes of insulation were observed in the loci of *COL24A1* and *FOXG1* (Supplementary Figure S3). Similar to the roles of RAD21 in regulating gene expression, as RAD21^R450C^ is co-expressed with wild-type RAD21 in affected LCLs, wild-type RAD21 may weaken the effects of RAD21^R450C^ and cause different profiles of insulation changes in these loci. This may explain the variant IS profiles within ISC bins in two affected-members LCLs (labeled with asterisks, Supplementary Figure S3). These results suggested that RAD21^R450C^ changed local contacts in these genes to alter their expression levels in the affected-members LCLs. In summary, our RNA-Seq and Hi-C analyses suggest that in cells expressing RAD21^R450C^, TAD boundaries near cell adhesion gene clusters are modulated, which correlates with gene expression changes in LCLs isolated from family members affected by peripheral sclerocornea.

### 2.3. Disrupted Neural Crest Migration in rad21^mut^-Injected X. laevis Embryos

**Figure 3:**
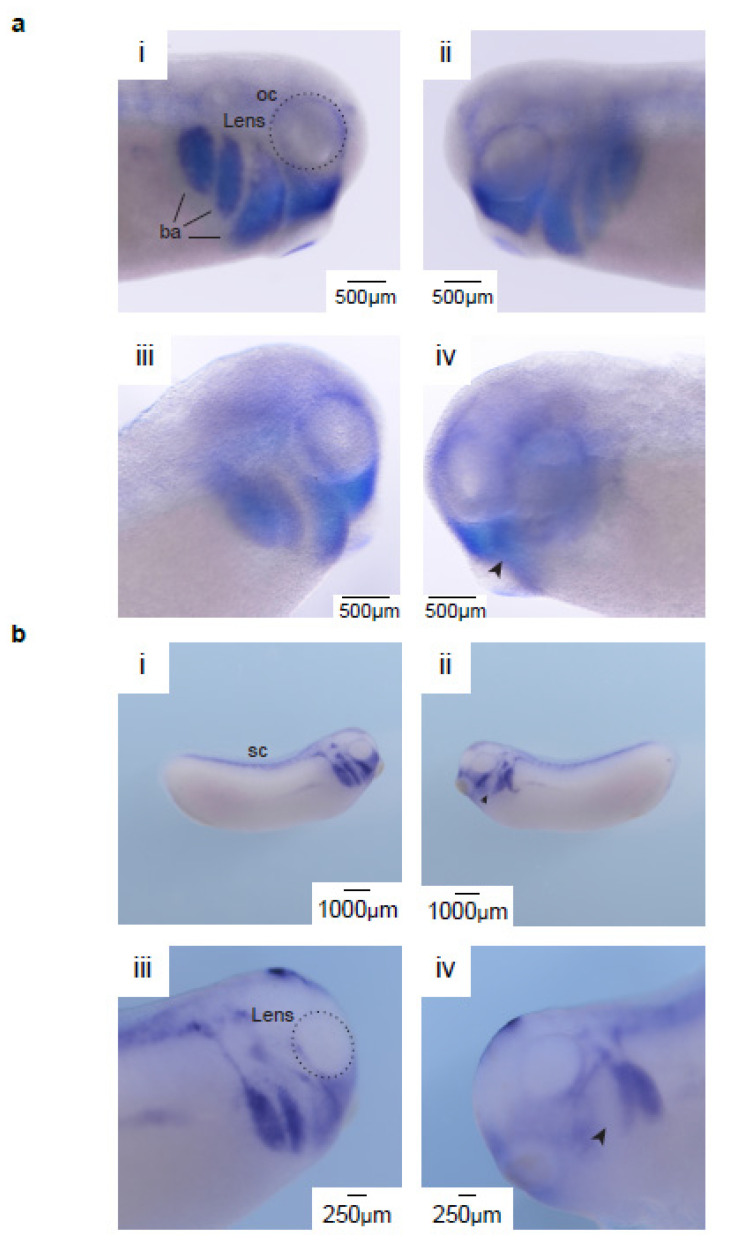

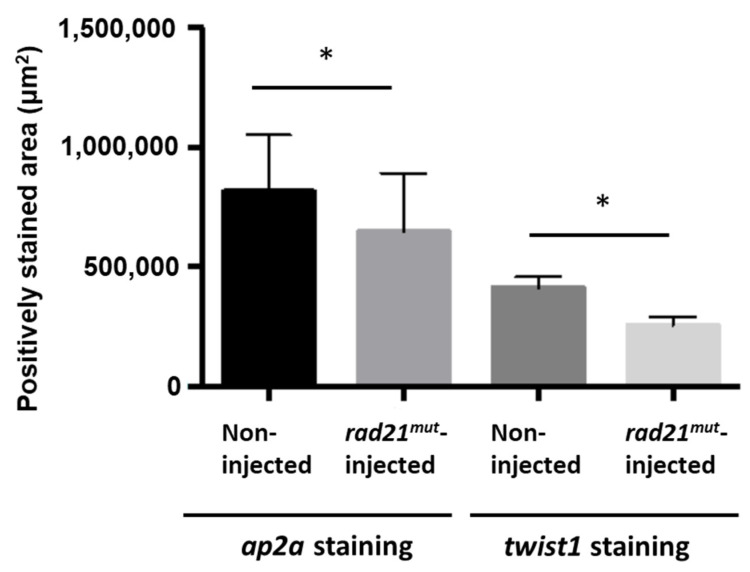
*rad21^mut^* disrupts the migration of neural crest cells (NCCs) in stage-25 *Xenopus laevis*. (**a**) The in situ hybridization pattern of *twist1* was altered in the *rad21^mut^*-injected side. Panel i and ii show *twist1* expression on both sides of the same non-injected embryo. The patterns around the optic cup (oc) and the neural crest migration streams into branchial arches (ba) are intact (indicated by a dash circle and dark solid lines, respectively). Panel iii and iv show the non-injected side and the injected side of the same *rad21^mut^*-injected embryo. The optic cup and branchial arch in panel iii are similar to those in panel i and ii, whereas these patterns are disrupted in panel iv. A total of 20 *rad21^mut^*-injected embryos were used, and 18 of them showed disrupted patterns in the *rad21^mut^*-injected side (18/20). (**b**) The in situ hybridization pattern of *ap2a* is altered in *rad21^mut^*-injected *X. laevis* embryos. Panel i shows *ap2a* is highly expressed in the head region and the spinal cord (sc) in the non-injected side. Panel ii shows the *ap2a* expression pattern is disrupted in the *rad21^mut^*-injected side. A total of 11 *rad21^mut^*-injected embryos were used, and 9 showed disrupted staining patterns in the *rad21^mut^*-injected side (9/11, arrowhead). Panel iii and iv show higher magnifications of the non-injected side and *rad21^mut^*-injected side of another embryo. Similar to panel ii, disrupted neural crest migration streams are shown in panel iv. A total of 11 *rad21^mut^*-injected embryos were used, and 9 showed disrupted staining patterns in the *rad21^mut^*-injected side (9/11, arrowhead). (**c**) Quantification of the mandibular areas showing positive staining of *ap2a* and *twist1*. Means and standard deviations for three injected sides were analyzed in each group. Statistics was done by using paired *t* test; * indicates *p* < 0.05.

Our RNA-Seq and Hi-C results indicated that RAD21^R450C^ upregulates the expression levels of cell adhesion genes, suggesting this RAD21 variants may alter cell migration during tissue morphogenesis [33]. In our previous study, we dissected the eyes of *X. laevis* embryos after microinjection of *LacZ* mRNA, wild-type mRNA, wild-type and mutant mRNAs and observed that disorganization of corneal collagens was induced by the mutant *rad21* but not by mechanical puncture [2]. To test whether RAD21^R450C^ altered cell migration and caused defects during cornea development, we overexpressed by microinjection the orthologous *rad21* mRNA carrying the same detected human mutation (*rad21^mut^*) and examined the migration of neural crest cells during *X. laevis* eye development. Neural crest cells originate from the neural plate border at the neurula stage and start migration as a cell mass in three segments before neural tube closure [9]. To determine whether *rad21^mut^* influenced neural crest cells migration, we stained for two neural crest markers, *twist1* and *ap2a*, using *X. laevis* embryos at stage 25 [34]. Figure 3a,b shows the staining of *twist1* and *ap2a*, respectively. Panel i and ii in Figure 3a show both sides of a non-injected embryo, and panel iii and iv show the non-injected and *rad21^mut^*-injected sides, respectively, of another embryo. Panel i, ii and iii show three major branchial arches (ba) and a clearly defined mandibular stream of NCC around optic vesicles. However, these structures were disrupted in the *rad21^mut^*-injected side (Figure 3a and panel iv), with a loss of NCCs entering the branchial arches as well as a not clearly defined mandibular stream. In total, this structure loss was observed in 18 of 20 (90%) injected sides of the *rad21^mut^*-injected embryos. The expression of *ap2a* was also disrupted upon overexpression of *rad21^mut^* (Figure 3b). We found that 9 of 11 (82%) *rad21^mut^*-injected embryos showed disruption of the NCC staining pattern (Figure 3b, panel ii and iv). We quantified the mandibular areas with positive staining of *ap2a* and *twist1*. Our results showed that for both *ap2a* and *twist1* staining, the *rad21^mut^*-injected side showed significantly smaller positively stained areas than the non-injected side (Figure 3c). We then injected *rad21^wt^* to rescue the disrupted NCC migration induced by *rad21^mut^*. Both *twist1* and *apa2* staining at stage 24 suggested that *rad21^wt^* could partially rescue the *rad21^mut^*-disrupted NCC staining patterns (Supplementary Figure S4). These results showed that *rad21^mut^* indeed altered NCC migration.

### 2.4. Decreased Periocular Mesenchymes Invading into the Cornea in rad21^mut^-Injected X. laevis Embryos

**Figure 4:**
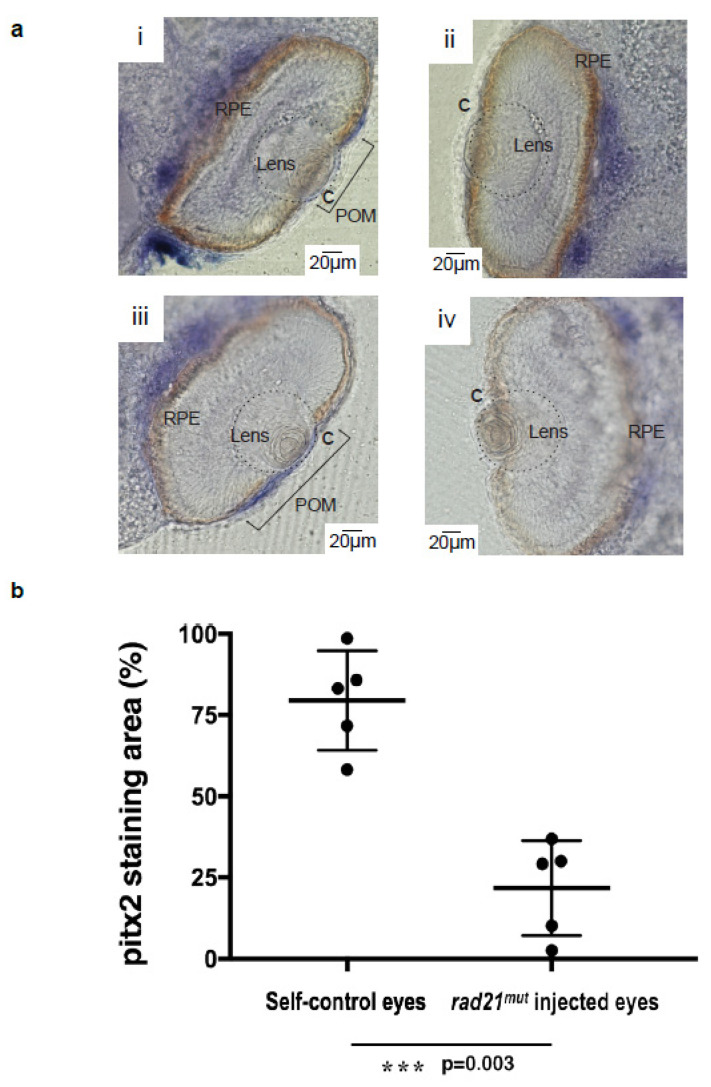
The expression of *pitx2* was decreased by injecting *rad21^mut^* into *X. laevis* embryos. The transcription factor *pitx2* is highly abundant in periocular mesenchymes (POM) and was used as a marker of POM. The lens is indicated by a dash circle. (**a**) Panel i and iii show the in situ hybridization expression pattern of *pitx2* (blue) in the non-injected side of two *X. laevis* embryos at stage 41. The other sides of all five embryos (5/5) injected with *rad21^mut^* mRNAs showed a lack of *pitx2* staining in the cornea (C) area (panel ii and iv). Normal structures of lens and retina layers could be observed in both the non-injected and the *rad21^mut^*-injected sides. (**b**) The quantification of *pitx2*-positive areas in the non-injected control eyes and the *rad21^mut^*-injected eyes was done using ImageJ. *** A significant decrease of staining corresponding to 57.7% was detected (*p* = 0.003).

Proper cell migration of periocular mesenchymes (POM) is essential for the formation of corneal stroma and keratocytes [35]. Disrupted mesenchymal migration is able to cause eye anterior segment diseases including sclerocornea [36,37]. POM are derived from cranial neural crest cells (CNCs) [38]. As neural crest cell migration during early development was disrupted after *rad21^mut^* injection, we further investigated whether mesenchymes entering the corneal region were also affected. A POM-specific marker, *pitx2*, was analyzed by staining during mesenchymes invasion into the corneal matrix at *X. laevis* developmental stage 41 [11,39,40]. Panel i and iii in Figure 4a show the non-injected sides of two embryos; POMs are stained purple in the cornea regions. In panel ii and iv of Figure 4a, no strong staining signals are observed in the cornea regions, indicating interrupted POMs migration into the cornea in the *rad21^mut^*-injected sides. In all embryos examined, similar staining patterns were observed. We quantified staining in the cornea areas positive for *pitx2* and found that 79.5% of the whole cornea areas were *pitx2*-positive in the non-injected sides, while only 21.8% of the whole cornea areas were *pitx2*-positive in the *rad21^mut^*-injected sides (*p* = 0.003) (Figure 4b). Taken together, our results clearly demonstrate that the *rad21^mut^* interfered with POM invasion into the corneal stroma region, which could further lead to reduced generation of keratocytes and corneal stroma thinning.

## 3. Discussion

Previously, we identified a rare peripheral sclerocornea pedigree in our clinic. With the application of high-throughput genetic methods, a candidate heterozygous variant of *rad21* was found associated with the disease. Injection of *rad21* mRNA carrying this mutation caused defects in the eyes and corneal stroma of *X. laevis* embryos [2]. Moreover, we found that even though *rad21* is universally expressed in multiple tissues, during embryonic development, *rad21* expression is enriched mainly in the eye region [2]. Our previous results showed that *rad21* plays an essential role in cornea development and the R450C variant impairs this role. However, the molecular mechanism is unclear. In this study, we first performed a transcriptome analysis to identify cell adhesion genes, the *PCDHG* gene clusters, whose expression levels were upregulated in LCLs isolated from peripheral sclerocornea-affected family members. We then performed Hi-C analysis to show that the RAD21 mutation caused changes in the local chromosome organization of the *PCDH* genes. Our results strongly indicated that the RAD21 variant disrupts the chromosome structure and expression of *PCDH* genes, thus causing cell migration defects during corneal development.

The gene *rad21* was first discovered by screening radiation-sensitive (rad) mutants in *Schizosaccharomyces pombe* [41]. It was found that *rad21* is sensitive mainly to ionizing radiation [42], suggesting it plays a role in repairing DNA double-strand breaks (DSBs). RAD21 is also a subunit of the cohesin complex. This complex is highly conserved in eukaryotes and is essential for various processes involving chromosomes. Following DNA replication in S phase, sister chromatids remain physically attached to each other until mitosis. Cohesin is a ring-shaped protein complex that embraces both sister chromatids along the length of each chromosome [43]. There are several ways to remove cohesin from the chromosomes. One way is to cut RAD21 proteolytically after the arginine residues 172 and 450 by activated separase [44]. Mutations of the separase cleavage sites in RAD21 lead to defective mitosis in human cells [44], which display incomplete separation of sister chromatids and the presence of lagging DNA and DNA bridges connecting sister chromatids [45]. Cells from our pedigree carrying the RAD21^R450C^ mutation did not display severe malformations such as chromosomal separation defects, but the affected family members displayed cornea opacification. Therefore, we investigated the underlying mechanisms of RAD21^R450C^ leading to cornea opacity in this study.

As sclerocornea is a congenital disease, the pathogenesis of this disease can be elucidated through studying eye development. During eye development, corneal stroma forms from the surrounding mesenchyme [46]. Disrupted migration of neural crest cells into the cornea has been proposed to be related to sclerocornea [36,37]. Therefore, we investigated the migration of neural crest cell and POMs entering the cornea by staining for specific markers. Indeed, disruption of neural crest cell migration and a significant decrease in POM numbers were observed. In conclusion, our results suggest a defect of neural crest migration as a possible mechanism contributing to the rare ocular disease sclerocornea.

Since the RAD21 variant did not impair sister chromatin separation in our previous studies, we reasoned that this RAD21 variant may regulate the local chromosome organization (insulation score), affecting the expression of developmental genes. Based on this rationale, we integrated our RNAseq and Hi-C data to show that this RAD21 variant was able to affect the local chromosome organization and upregulate the expression of *PCDHG* gene clusters. Since *PCDHG* genes are critical for cell adhesion and migration, we further studied these genes and cell migration in *X. laevis* embryos. For genes coding for MHC class II, we did not observe any significant changes in their local chromosome organization. Therefore, we did not investigate the genes for MHC class II. The size of the mitochondrial genome is about 17 kb, which is below our Hi-C resolution (20 kb). We were thus unable to analyze whether this RAD21 variant affects the organization of the mitochondrial genome using Hi-C. In addition, it is unclear whether RAD21 can enter the mitochondria and regulate mitochondrial genome organization. Therefore, this RAD21 variant may exert an indirect effect on mitochondrial gene expression.

Our functional enrichment analyses suggested that cell migration properties could be affected by the *rad21* variant. Indeed, we could observe NCC migration defects in *rad21^mut^*-injected embryos. Our next aim was to establish the mechanistic details of the *rad21* variant effects on NCC migration. 

A fundamental characteristic of migrating NCCs is contact inhibition of locomotion (CIL) [47]. The ordered and directional migration of NCCs is dependent on CIL, which directs NCCs away from each other [47], separating them from placodal cells, an epithelial tissue that contributes to sensory organs, after their initial contacts [48]. There are two major molecular machineries regulating CIL: the cadherin switch generated by EMT [49] and the interactions between erythropoietin-producing human hepatocellular receptors (Eph) and Eph receptor-interacting proteins (Ephrin) [50,51]. Among the genes showing significant ISC in Hi-C datasets, three genes belong to the family of Eph receptors or ephrin ligands, namely, *EPHA3*, *EPHA5*, and ephrin A5 (*EFNA5*). Intriguingly, both receptors EphA3 and EphA5 show interactions with the ligand ephrin A5 during early embryonic development [52,53]. In addition, the expression of EphA3 can be detected in all three cell types present in the cornea [54], i.e., epithelial cells, keratocytes, and endothelial cells, whereas EphA5 cannot be detected in any of the cornea cell types or tissues [54]. Ephrin A5 has been reported to be highly expressed in mouse lens epithelial cells. Eph–ephrin signaling controls cell adhesion and migration by modifying the cytoskeleton and regulating adhesion-related molecules. Two opposite behaviors can be expected in cells interacting through Eph receptors and ephrins: attraction or repulsion [55,56]. During neural crest migration, repulsive effects play major roles to restrict the segmental migration of NCCs [57]. In the future, antagonists of Eph–ephrin interaction can be applied during embryo development to evaluate whether CNC migration can be restored.

## 4. Materials and Methods

### 4.1. Ethics

This study adhered to the tenets of the Declaration of Helsinki and was approved by the institutional review board of Kowloon Central Cluster, Hospital Authority, Hong Kong. Informed consent was obtained from participants with the peripheral sclerocornea pedigree for taking blood samples and subsequent work with cell lines derived from their blood. Animal ethics approval for this study was obtained from the Animal Ethics Committee of The Chinese University of Hong Kong and the Department of Health of the Hong Kong Special Administrative Region.

For animal experiments, we obtained the ethics approval from the Department of Health, the Government of the Hong Kong Special Administrative Region. The ethics approval was granted on 25 February 2016 with the approval number (15-970) in DH/HA&P/8/2/1 Pt.55.

For human experiments, we obtained the ethics approval from the institutional review board of Kowloon Central/Kowloon East Cluster, Hospital Authority, Hong Kong. The ethics approval was granted on 27 January 2016 with the approval number (KC/KE-15-0223/ER-1).

### 4.2. Lymphoblastoid Cell Lines

LCLs were established and cultured as previously reported [5]. Briefly, whole blood was taken from sclerocornea pedigree members, and the peripheral blood mononuclear cell layer was isolated and transformed with B98-5 Epstein–Barr virus (EBV; VR-1492, ATCC, Manassas, VA, USA).

### 4.3. RNA Sequencing

Total RNA of LCLs derived from pedigree members II-2, II-3, II-4, II-5, and III-5 was extracted and purified. A cDNA library was constructed by Macrogen (Seoul, Korea) using the Illumina TruSeq RNA sample preparation kit v2 (Illumina, Hayward, CA, USA). RNA-Seq was conducted on Illumina HiSeq 2500. For sequencing quality assessment, GC content, Q20 percentage, and Q30 percentage were examined. Raw sequencing reads were processed and analyzed in our collaborator’s, Job Dekker, lab following their previously reported protocol [58]. The list containing all protein-coding genes and their reads per kilobase million (RPKM) were generated by the Dekker’s lab. The fold change (FC) of RNA abundance in LCLs between affected and unaffected members was calculated as the two RPKM values of the two affected members, II-2 and II-3, divided by the two RPKM values of the two unaffected members, II-4 and II-5. The FC was further log-transformed. The mean and the standard deviation of all Log_2_FC were calculated, and the 95% confidence interval (CI) was calculated accordingly. Gene ontology enrichment analysis was conducted using DAVID (https://david.ncifcrf.gov/summary.jsp) and Enrichr (http://amp.pharm.mssm.edu/Enrichr/). The heatmap was generated using R gplots.

### 4.4. Quantitative Real-Time PCR (qPCR)

Complementary DNA (cDNA) was synthesized with the SuperScript III First-Strand Synthesis System (ThermoFisher Scientific, Waltham, MA, USA) and oligo (dT)_20_. Expression of candidate genes for RNA-Seq validation and mesenchymal marker genes was analyzed using the SYBR Green method (LightCycler 480 SYBR Green I Master, Roche, Indianapolis, IN, USA) and the LightCycler 480 II (Roche, Indianapolis, IN, USA). Primers are list in Supplementary Table S6.

### 4.5. Hi-C

Hi-C was performed as previously described [59]. Briefly, LCLs (5 × 10^6^) were cross-linked in 1% formaldehyde for 10 min and then quenched in 125 mM glycine. The fixed cells were shipped to Job Dekker’s lab for further processing and analysis. Cross-linked cells were lysed in lysis buffer (10 mM Tris-HCl pH 8.0, 10 mM NaCl, 0.2% Igepal CA-630, and Halt protease inhibitors Thermo Fisher 78429). After disruption, chromatin was solubilized and digested using 400 units of DpnII at 37 °C overnight. DNA overhangs were then filled in with 0.4 mM biotin-14-dATPs and ligated with 50U T4 ligase at 16 °C for 4 h. Cross-links of ligated DNAs were reversed with 10 mg/mL proteinase K (Life Technologies, 25530-031, Waltham, MA, USA) at 65 °C overnight, and DNA was purified using phenol/cholorform. After the removal of biotin from the unligated ends, DNA was fragmented to 150–350 bps using an E220 sonicator (Covaris, Woburn, MA, USA). After end-repair, biotinylated DNA was enriched using streptavidin beads (MyOne C1 beads, Life Technologies, 650.01, Waltham, MA, USA). Hi-C libraries were generated by using the Illumina TruSeq Nano DNA kit (Illumina, Hayward, CA, USA). Hi-C libraries were sequenced on an Illumina HiSeq-4000 (Illumina, Hayward, CA, USA), and raw sequencing data in the Fastq format were obtained.

Hi-C data were processed according to a previous published method [60]. Fastq files were mapped and binned using the c-World pipeline from the Dekker’s lab, which is available at a GitHub repository [61] (https://github.com/dekkerlab/cMapping; https://github.com/dekkerlab/balance; https://github.com/dekkerlab/cworld-dekker). Briefly, 50 bp paired-end reads were truncated to 25 bp starting at the 5-prime and then were iteratively mapped onto hg19 human reference genome. Uniquely mapped paired-end reads were collected and assigned to Dpn II restriction fragments based on their 5-prime locations. Mapped reads with same fragment ends and uniqueness were kept, and PCR duplicates were removed. Interaction heat maps, insulation scores, and loop pile-ups were generated using scripts included in the c-World pipeline. The loop coordinates used for loop pile-up analysis were obtained from GM12878 cells [62]. The ISC for LCLs from unaffected and affected members was calculated using 20 kb binned Hi-C data and a 500 kb insulation square window. The coding genes having ISC bins were identified with Bedtools.

### 4.6. Wild-Type and Mutant X. laevis Embryos

Wild-type *X. laevis* embryos were microinjected with mutant *rad21* and *LacZ* mRNAs as previously reported [2]. Embryos were collected for further staining and histology investigation.

### 4.7. Whole-Mount In Situ Hybridization (WISH)

Digoxigenin-labeled RNA probes used for in situ hybridization were synthesized with the primers listed in Supplementary Table S6. PCR primers were designed to amplify parts of the target gene. On the reverse primer, a T7 promoter sequence (5′ TAA TAC GAC TCA CTA CTA TAG GG 3′) was added. After purification, the amplicon was ready for in vitro transcription using the same protocol as for the linearized plasmid. The staining procedure was done according to a previously published protocol [2]. After in situ hybridization with the antisense probes of *ap2a* or *twist1*, their staining areas in mandibular crest stream were measured by using the NIS-Elements BR software (Nikon, Melville NY, USA).

### 4.8. Vibratome Section

After in situ hybridization, vibratome sectioning of the stained embryos was conducted to detect the signal. Embryos were washed with PBS twice, then 1 part of 25% glutaraldehyde was evenly added to 9 parts of gelatin/albumin embedding medium (22.5 mL 10 × PBS, 1.1 g gelatin, and 67.5 g albumin in 225 mL water). The mixture was stirred quickly and then applied to a plastic mold. Each embryo was transferred into a mold to stand at a right orientation with the cutting surface facing the bottom of the mold. The molds containing the samples were incubated on ice to solidify. The blocks were then trimmed smaller to fit in the metal block of the vibratome machine (Leica, Wetzlar, Germany). Sections were prepared with 50 µm thickness. The target sections were mounted on slides for microscopy investigation. Paired-t test was used to compare the measured areas with *pitx2* staining in normal control eyes and *rad21^mut^*-injected eyes.

## Supplementary Materials

Supplementary materials can be found at https://www.mdpi.com/1422-0067/21/20/7807/s1. Figure S1: The top six up- and top four down-regulated genes from the RNA sequencing results were selected for qPCR validation; Figure S2: Global chromosome organization of LCLs; Figure S3: Local chromosome structural changes in two differentially expressed genes; Figure S4: Rescue of disrupted *twist1* and *apa2* expression patterns; Table S1: Coding genes that were differentially expressed between LCLs isolated from affected and unaffected family members; Table S2: Clusters of genes that were differentially expressed in LCLs isolated from affected family members; Table S3: ISC between the affected and unaffected LCLs; Table S4: Significant enrichment of ISCs in significant E1Cs; Table S5: Significant ISC bins overlapping with coding gene loci; Table S6: Primer sequences used in this study.
