# Supplementary material for: rad21 Is Involved in Corneal Stroma Development by Regulating Neural Crest Migration
Source: Int J Mol Sci. 2020 Oct 21;21(20):7807. doi: 10.3390/ijms21207807 (PMC7594026; doi:10.3390/ijms21207807)
Supplement: Supplementary file 1 [file ijms-21-07807-s001.zip › Supp Figure S1.pdf]

Affected (2 cell lines) relative to unaffected (3 cell lines)				
RNAseq			Taq-Man qPRC	
**Gene name**	**Regulation**	**Fold change (after Log2 processing)**	**Regulation**	**Fold change (after Log2 processing)**
SEPT10	Up	2.60	Up	2.94±0.03
PCDHGC3	Up	1.36	Up	0.81±0.11
RPS18	Up	0.87	Up	0.24±0.11
CCZ1	Up	1.17	Up	0.3±0.06
SLFN12L	Up	1.06	Up	1.82±0.53
RPS3A	Up	0.71	Up	0.82±0.63
GTSF1	Down	-3.84	Down	-4.5±1.13
IL32	Down	-2.08	Down	-1.4±0.73
ARHGAP44	Down	-2.40	Down	-1.09±0.61
GOLGA8B	Down	-1.49	Down	-1.43±0.3
