# Supplementary material for: rad21 Is Involved in Corneal Stroma Development by Regulating Neural Crest Migration
Source: Int J Mol Sci. 2020 Oct 21;21(20):7807. doi: 10.3390/ijms21207807 (PMC7594026; doi:10.3390/ijms21207807)
Supplement: Supplementary file 1 [file ijms-21-07807-s001.zip › Supplementary Table S6.docx]

**Supplementary Table S6.** List of primers.

| Gene | Forward Primer (5'→3') | Reverse Primer (5'→3') |
| --- | --- | --- |
| ***For in situ hybridization in X. laevis*** | | |
| *pitx2 _S* | GCTGCAGGAGCTGGAAG | TAATACGACTCACTATAGGGAGAGGCTGCATCAGTCCATTG |
| ***For RNA-Seq validation*** | | |
| *SEPT10* | CCAGTGATTGCCAAAGCAG | CCGACTTTTACCTCATCCATAC |
| *PCDHGC3* | CCGATGTGGGAAGCAACTC | CGCGTCCAGCACCTTG |
| *RPS18* | CAACATCGATGGGCGGC | CCCGCCCTCTTGGTGAG |
| *CCZ1* | CATGAGTGCGGCTGTGTG | GGCGAGATTCATGTGGTTGAAG |
| *SLFN12L* | GGAGCTTGGAAACCTCTGGTC | GCCAAGGCTTCCATGTTACTTTC |
| *RPS3A* | GGCATGGATCTTACCCGTG | GTCTGCACCTCTCGGGTC |
| *GTSF1* | GGAAGAAACTTACACCGACTCC | GGCTCCTGGTTTGGTTGAC |
| *IL32* | CTGGGGAGAGCTTTTGTGAC | GGGGTTCAGAGCACTTCTG |
| *ARHGAP44* | GGTGGCAGCAGACTTCC | CAGGCCTCCTGTTGTGC |
| *GOLGA8B* | GGGTTCTCGAGCCGCAG | CCACTGGGCCCTCTCTC |
