# Supplementary figures and images for: rad21 Is Involved in Corneal Stroma Development by Regulating Neural Crest Migration
Source: Int J Mol Sci. 2020 Oct 21;21(20):7807. doi: 10.3390/ijms21207807 (PMC7594026; doi:10.3390/ijms21207807)

**a**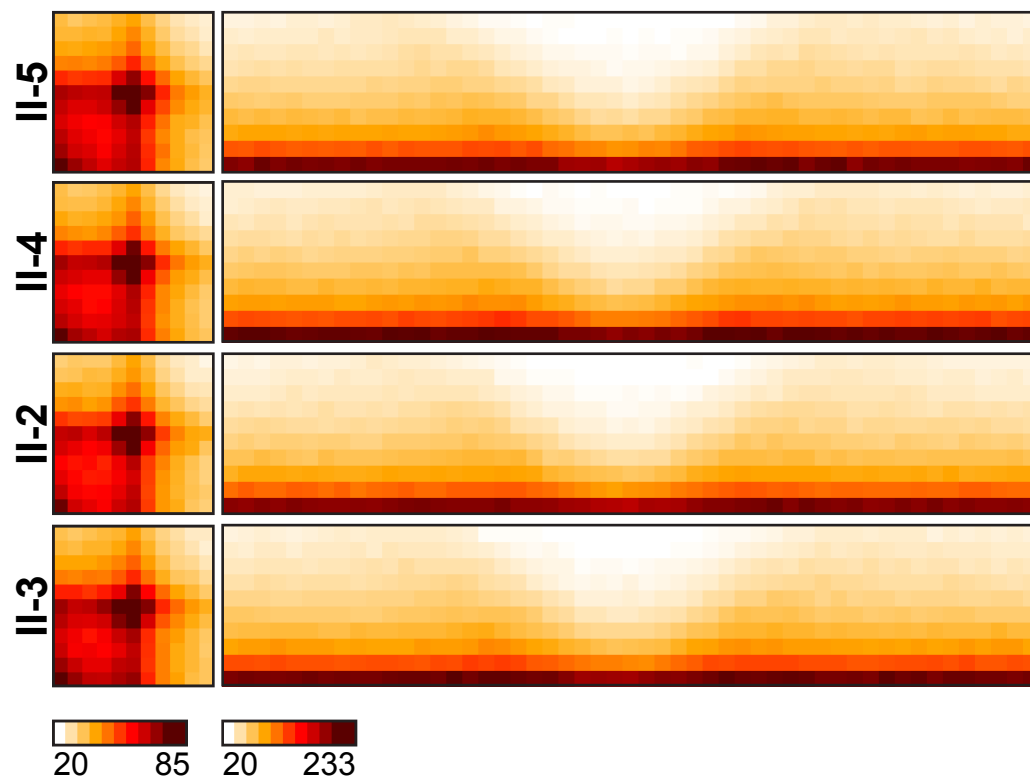**b**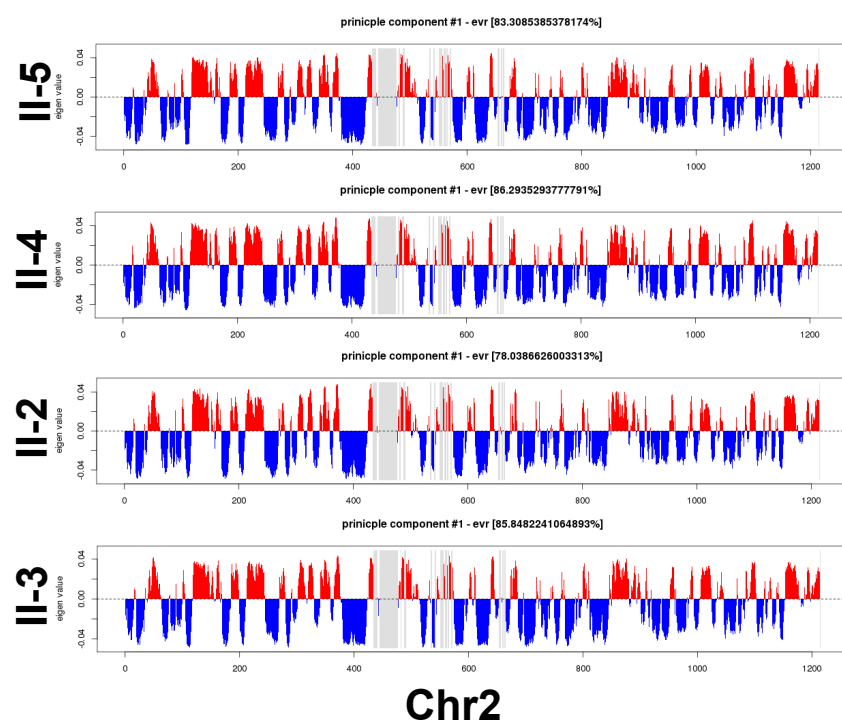

Supplement: Supplementary file 1 [file ijms-21-07807-s001.zip › Supple Figure S2.pdf]

**a**

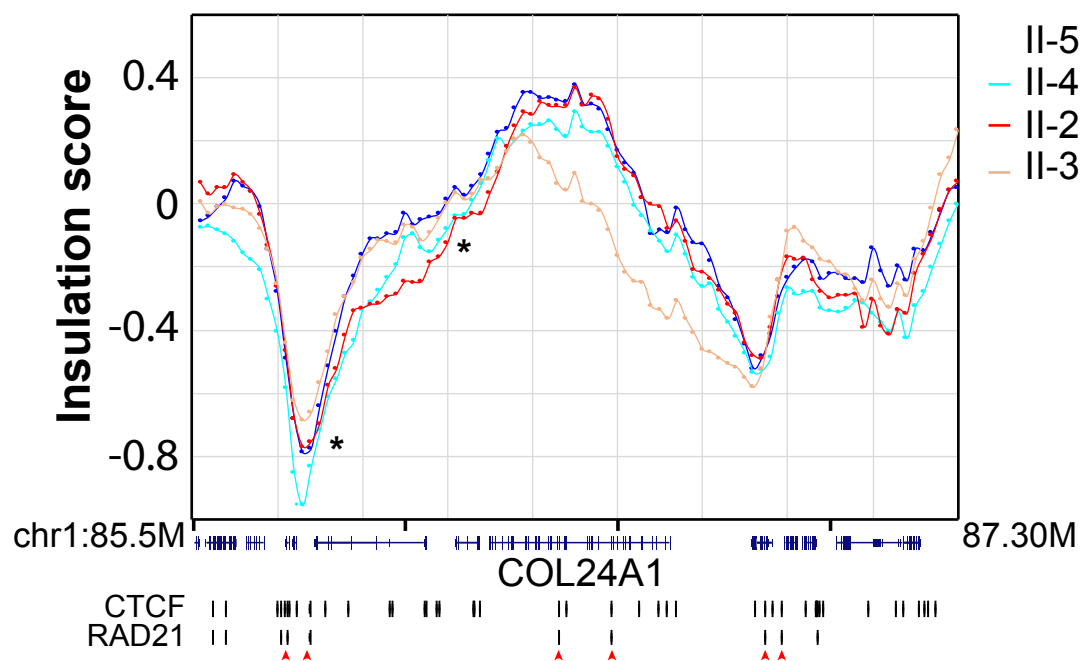

**b**

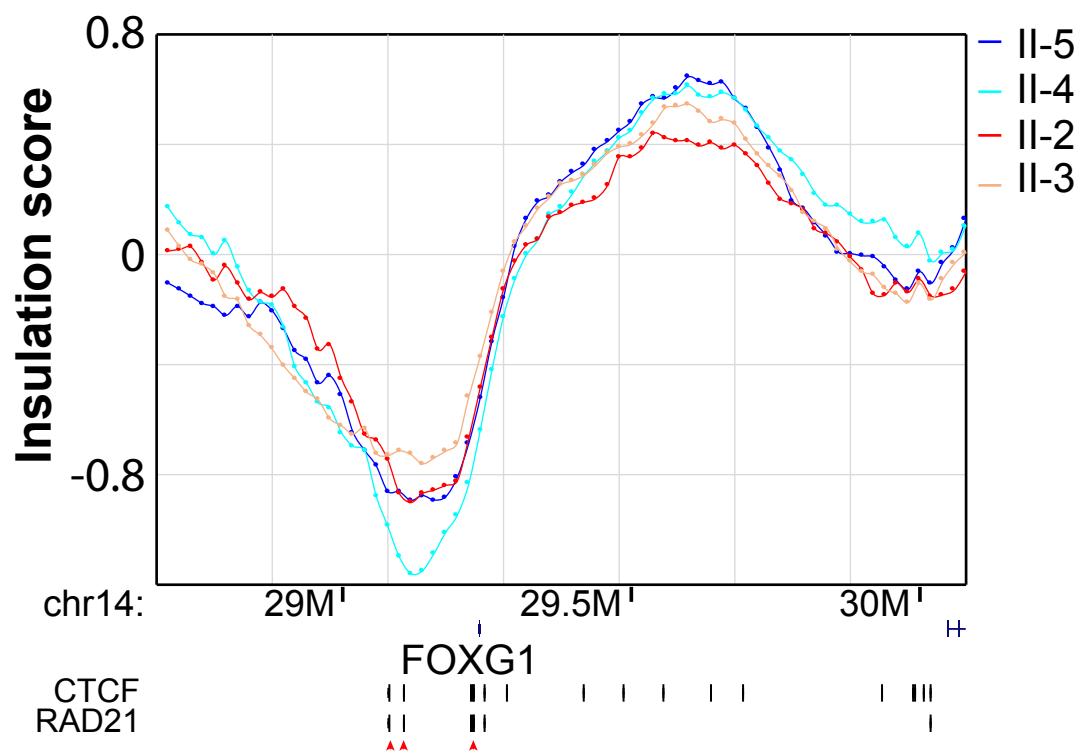

Supplement: Supplementary file 1 [file ijms-21-07807-s001.zip › Supple Figure S3.pdf]

**a**

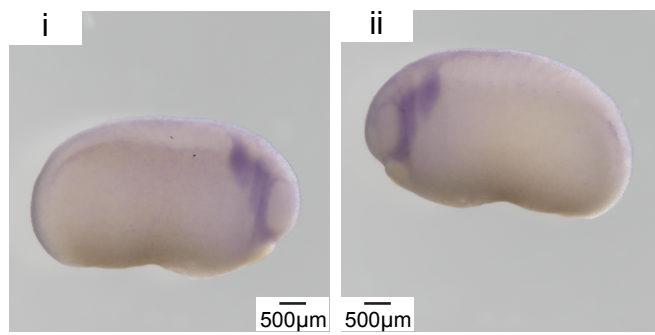

**b**

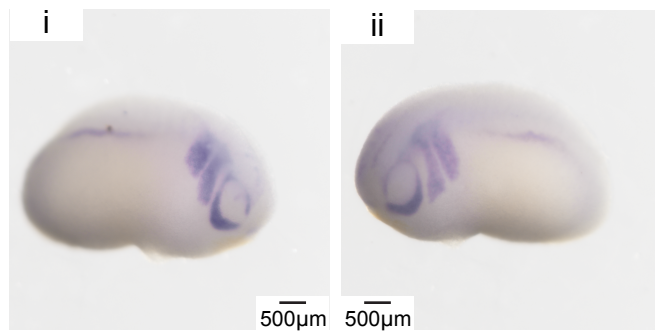

Supplement: Supplementary file 1 [file ijms-21-07807-s001.zip › Supple Figure S4.pdf]
